# WORKING LIFE EXPECTANCY OF OLDER ADULTS: FINDINGS FROM ASIA AND THE AMERICAS

**DOI:** 10.1093/geroni/igae098.1416

**Published:** 2024-12-31

**Authors:** Rahul Malhotra, Abhijit Visaria

**Affiliations:** Chair; Co-Chair

## Abstract

With a growing focus on productive ageing, including engagement in work, it is important to assess its extent across key demographic sub-groups within a country, and if feasible, across countries. This will help identify disadvantaged sub-groups in the context of productive ageing. Working life expectancy (WLE; years of remaining life one can expect to work at a given age), a population-level indicator of productive ageing, allows one to capture variation in the duration of working life due to societal, legislative, and economic considerations. The four presentations in this symposium provide empirical findings on WLE from nationally representative longitudinal studies of middle-aged and/or older adults from countries in Asia or the Americas. The first, from Japan, projects WLE till 2050 and highlights the role of policy interventions in increasing it. The second, from Mexico, observes strong sex disparity in WLE, underscoring the need for policies to promote women’s work participation. The third from Chile, where mandatory retirement age is lower for women, finds disparity in WLE at retirement age not by sex but by working status, highlighting mandatory retirement age’s influence on WLE. The fourth from Singapore, using data from two longitudinal cohorts, observes maintenance of sex disparity (disfavoring women) but reversal of education disparity (disfavoring those with lower education) in WLE over time. Collectively, they highlight variability in WLE at older ages within and across countries, in terms of absolute or relative (to life expectancy) WLE, disparities in in WLE, and potential options for policy focus for increasing WLE.

